# *In Vitro* Gender-Dependent Inhibition of Porcine Cytochrome P450 Activity by Selected Flavonoids and Phenolic Acids

**DOI:** 10.1155/2015/387918

**Published:** 2015-01-21

**Authors:** Bo Ekstrand, Martin Krøyer Rasmussen, Felicia Woll, Vladimir Zlabek, Galia Zamaratskaia

**Affiliations:** ^1^Department of Food Science, Aarhus University, 8830 Tjele, Denmark; ^2^Department of Food Science, BioCenter, Swedish University of Agricultural Sciences, P.O. Box 7051, 750 07 Uppsala, Sweden; ^3^South Bohemian Research Center of Aquaculture and Biodiversity of Hydrocenoses, Faculty of Fisheries and Protection of Waters, University of South Bohemia in Ceske Budejovice, 389 25 Vodnany, Czech Republic

## Abstract

We investigated gender-related differences in the ability of selected flavonoids and phenolic compounds to modify porcine hepatic CYP450-dependent activity. Using pools of microsomes from male and female pigs, the inhibition of the CYP families 1A, 2A, 2E1, and 3A was determined. The specific CYP activities were measured in the presence of the following selected compounds: rutin, myricetin, quercetin, isorhamnetin, *p*-coumaric acid, gallic acid, and caffeic acid. We determined that myricetin and isorhamnetin competitively inhibited porcine CYP1A activity in the microsomes from both male and female pigs but did not affect the CYP2A and CYP2E1. Additionally, isorhamnetin competitively inhibited CYP3A in both genders. Noncompetitive inhibition of CYP3A activity by myricetin was observed only in the microsomes from male pigs, whereas CYP3A in female pigs was not affected. Quercetin competitively inhibited CYP2E1 and CYP1A activity in the microsomes from male pigs and irreversibly CY3A in female pigs. No effect of quercetin on CYP2E1 was observed in the microsomes from female pigs. Neither phenolic acids nor rutin affected CYP450 activities. Taken together, our results suggest that the flavonoids myricetin, isorhamnetin, and quercetin may affect the activities of porcine CYP1A, CYP3A, and CYP2E1 in a gender-dependent manner.

## 1. Introduction

Hepatic Phase I and II metabolism is the major pathway for the biotransformation of xenobiotic compounds. The cytochrome P450 family (CYP450) is the main group of Phase I enzymes, contributing to the metabolism of various endogenous and xenobiotic compounds [1]. Observed differences between individuals in activities of CYP450 depend on numerous factors including genetic profile, health status, and dietary intake. Moreover, it is known that CYP450 expression and activity can vary between genders [2]. Only a limited number of studies have compared regulation of catalytic activity of individual CYP450 between males and females, giving the impression that gender differences are more pronounced in animal models [3]. With the increasing acceptance of porcine CYP450 as a useful and important model for human CYP450, remarkably few studies have investigated this phenomenon in pigs. We have previously suggested that the regulation of specific porcine CYP450 activity is different between genders [4, 5]. These studies showed that treatment with steroids or an extract of chicory root modified CYP450 activity differently in microsomes from male and female pigs.

Both* in vivo *and* in vitro* dietary flavonoids have been demonstrated to modulate human CYP450s [6, 7]. In fact, a number of studies have demonstrated that flavonoids are potent inhibitors of various CYP450 isoforms and membrane transporters and thus interact with pathways of many commonly prescribed drugs [8, 9]. Hence, knowledge on regulation of CYP450-dependent metabolism by this group of compounds is highly relevant in both humans and animals, as they are both exposed to drugs and other xenobiotics. However, to our knowledge, no studies have investigated whether the effect of flavonoids differs between genders.

In the present study, we investigated the hypothesis that the effects of selected flavonoids on CYP450 activity are dependent on gender. Thus, the ability of selected flavonoids and phenolic acids to interfere with the activity of porcine CYP1A, CYP2A, CYP2E1, and CYP3A was studied using microsomes from male and female pigs raised under similar conditions.

## 2. Materials and Methods

### 2.1. Animals and Sampling

All animals included in this study were treated in accordance with the guidelines from the Danish Inspectorate of Animal Experimentation. In total, 6 male and 4 female pigs were used in this study. All pigs were crossbreeds between Landrace X Yorkshire and Duroc, subjected to the same feeding regime, slaughtered at the same age, and kept under the same conditions. Details are given by Rasmussen et al. [4, 10].

### 2.2. Chemicals and Reagents

Gallic acid, caffeic acid,* p*-coumaric acid, rutin, and quercetin were purchased from Sigma Aldrich (St. Louis, MO); myricetin and isorhamnetin were from Fluka (Buchs, Switzerland). Stock solutions of flavonols and phenolic acids were prepared in methanol and were stored at −80°C. HPLC grade acetonitrile and methanol were purchased from Merck (Darmstadt, Germany).

### 2.3. Hepatic Microsome Preparation

Microsomes were prepared with a calcium aggregation method using Tris-EDTA homogenization buffer [11]. Experiments were performed using pools of microsomes consisting of microsomes from two individual pigs. The total microsomal protein concentration was analyzed the same day using a commercially available kit (Bio-Rad Laboratories Inc., Hercules, CA, USA) according to the manufacturer's instructions. Microsomes were diluted to a protein concentration of 10 mg/mL and stored at −80°C until analysis.

### 2.4. Measurement of Specific CYP450 Activity

Specific CYP450 activities were determined by incubating microsomes (diluted in 0.5 M potassium phosphate buffer (pH 7.4)) and adding 0.5 mM NADPH together with one of the following selective substrates (specific CYP450 target given in parentheses): 2 *μ*M 7-ethoxyresorufin (CYP1A), 200 *μ*M coumarin (CYP2A), 10 *μ*M 7-benzyloxy-4-trifluoromethylcoumarin (CYP3A), and 200  *μ*M* p*-nitrophenol (CYP2E1). For kinetic studies, concentrations of 7-ethoxyresorufin varied from 0.05 to 4 *μ*M, concentrations of* p*-nitrophenol varied from 2.5 to 4000 *μ*M, and concentrations of BFC varied from 0.1 to 150 *μ*M. The corresponding metabolites were quantified by HPLC as previously described [12–14].

### 2.5. Inhibition Assays

The CYP450 activity was evaluated in the presence of 16 *μ*M of the following compounds: rutin, myricetin, quercetin, isorhamnetin,* p*-coumaric acid, gallic acid, and caffeic acid. For quercetin, this concentration is approximately what can be found in liver of pigs fed a single dose of a quercetin enriched diet [15]. The stock solutions of these compounds were prepared in methanol, and the final concentration of methanol was 0.1% in the incubation volumes. The same amount of methanol was added to the control incubations. Percentage inhibitions were calculated as differences between the activities in the control incubations (arbitrarily set to 100%) and in the presence of the specific compound.

For compounds which inhibited the specific CYP450 activity by at least 20% when used in 16 *μ*M concentration, the mode of inhibition was determined. Moreover, in order to distinguish between reversible and irreversible inhibition, a preincubation step was included. For that microsomes were preincubated with the tested compound in the presence of NADPH for 5 min (CYP1A, CYP3A) or 15 min (CYP2E1) at 37°C before the addition of specific substrate. The choice of preincubation time was based on our previous experiment and was shown to cause the highest ability of test compound to modify enzyme activity. In this experimental set, a single concentration of the substrate and three concentrations of inhibitor were used (16, 32, and 128 *μ*M). An inhibition mode of reversible inhibition (competitive or noncompetitive) was further determined using the same concentrations of inhibitor (16, 32, and 128 *μ*M) and 6 or 8 substrate concentrations. CYP1A activity was determined at 7-ethoxyresorufin concentrations from 0.1 to 4 *μ*M, CYP3A activity was determined at BFC concentrations from 0.1 to 150 *μ*M, and CYP2E1 activity was determined at* p*-nitrophenol concentrations from 0.25 to 4000 *μ*M. The incubation time and the amount of the microsomal protein were in the linear range for the rate of metabolite formation. All measurements were performed in duplicate.

### 2.6. Data Analysis

Kinetic parameters (Michaelis-Menten constant *K*_*m*_, maximal velocity of the reaction *V*_max⁡_, and dissociation constant for inhibitor binding *K*_*i*_) were estimated by fitting data to the Michaelis-Menten equation using GraphPad Prism (version 4.0 for Windows, GraphPad Software (San Diego, California, USA)). Visual inspection of the plots was used to estimate whether inhibition degree was affected by inclusion of the preincubation step. If the magnitude of inhibition increased after preincubation compared to no preincubation, 50% inhibition of CYP450 activity (IC_50_ value) was determined by GraFit.

## 3. Results

**Figure 1:**
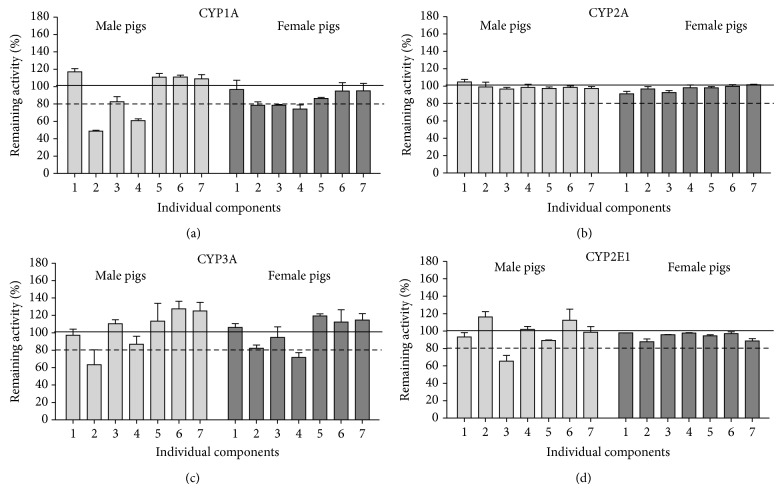
*In vitro* inhibition of (a) CYP1A, (b) CYP2A, (c) CYP3A, and (d) CYP2E1 by individual flavonols and phenolic acids (16 *μ*M) in hepatic microsomes from male and female pigs. The following compounds were used: 1, rutin; 2, myricetin; 3, quercetin; 4, isorhamnetin; 5,* p*-coumaric acid; 6, gallic acid; 7, caffeic acid. Data are presented as the mean percentage of remaining activity and standard error of the enzyme activity for two (for CYP1A, CYP2A) or three (for CYP3A, CYP2E1) individual pools with microsomes from 2 male pigs in each pool and two pools with microsomes from 2 female pigs in each pool.

**Figure 2:**
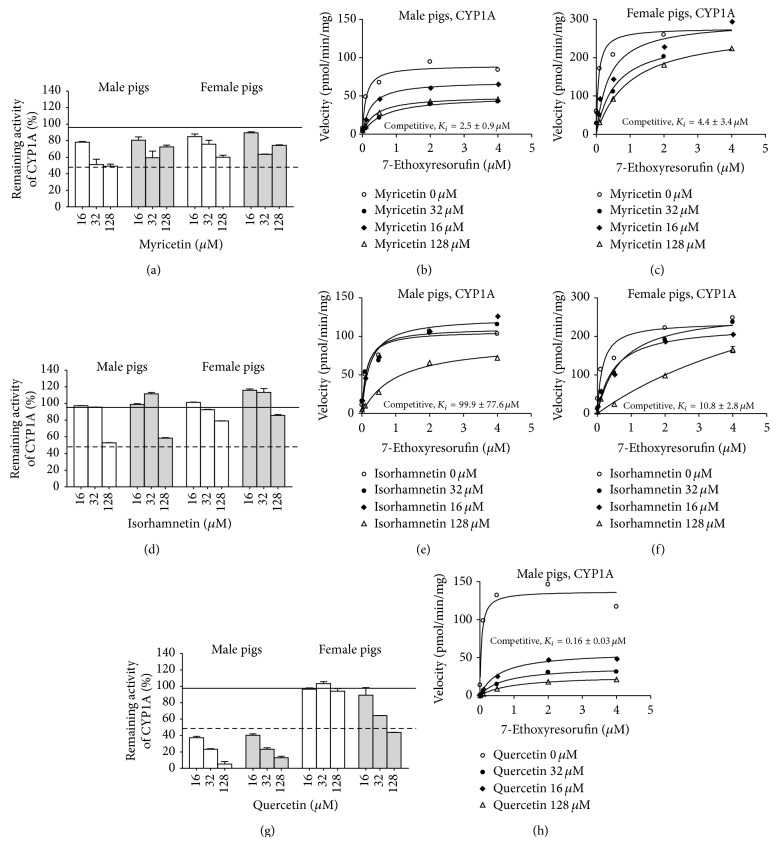
*In vitro* inhibition of CYP1A by myricetin, isorhamnetin, and quercetin in hepatic microsomes from male and female pigs. Data are presented as the mean percentage of remaining activity and standard error of the enzyme activity for three pools with microsomes from 2 male pigs in each pool and two pools with microsomes from 2 female pigs in each pool. (a) Effect of myricetin with (grey bars) and without (white bars) 5 min preincubation. ((b) and (c)) Saturation curve for 7-ethoxyresorufin O-deethylation in hepatic microsomes from male (b) or female (c) pigs in the presence of myricetin. (d) Effect of isorhamnetin with (grey bars) and without (white bars) 5 min preincubation. ((e) and (f)) Saturation curve for 7-ethoxyresorufin O-deethylation in hepatic microsomes from male (e) or female (f) pigs in the presence of isorhamnetin. (g) Effect of quercetin with (grey bars) and without (white bars) 5 min preincubation. (h) Saturation curve for 7-ethoxyresorufin O-deethylation in hepatic microsomes from male pigs in the presence of quercetin.

Of the tested compounds, myricetin, quercetin, and isorhamnetin decreased CYP1A activity measured as the metabolism of 7-ethoxyresorufin (Figure 1(a)). For myricetin and isorhamnetin, this reduction was more pronounced in microsomes from male pigs (remaining activity in the presence of myricetin from 44.9 to 50.1% in male and from 70.3 to 85.9% in female pigs; in the presence of isorhamnetin from 57.3 to 66.0% in male and from 65.7 to 85.9% in female pigs). Quercetin inhibited CYP1A activity in the microsomes from both male and female pigs to a similar degree (remaining activity from 70.7 to 74.6% in male and from 77.7 to 79.4 in female pigs). Based on these results, we further investigated the inhibitory effect of myricetin, quercetin, and isorhamnetin on CYP1A activity. Including a preincubation step did not affect inhibition of CYP1A by either myricetin or isorhamnetin (Figures 2(a) and 2(d)). Analysis of the inhibition mode indicated that both compounds acted as competitive inhibitors of CYP1A activity, with *K*_*i*_ values being lower in female pigs (Figures 2(b), 2(c), 2(e), and 2(f)). For quercetin, the inclusion of a preincubation step increased the magnitude of inhibition of CYP1A activity in microsomes from female pigs indicating irreversible inhibition (IC_50_ = 2.5 *μ*M, Figure 2(g)). In male pigs, quercetin inhibited CYP1A activity in a competitive manner (Figure 2(h)).

None of the tested compounds affected CYP2A activity (Figure 1(b)).

**Figure 3:**
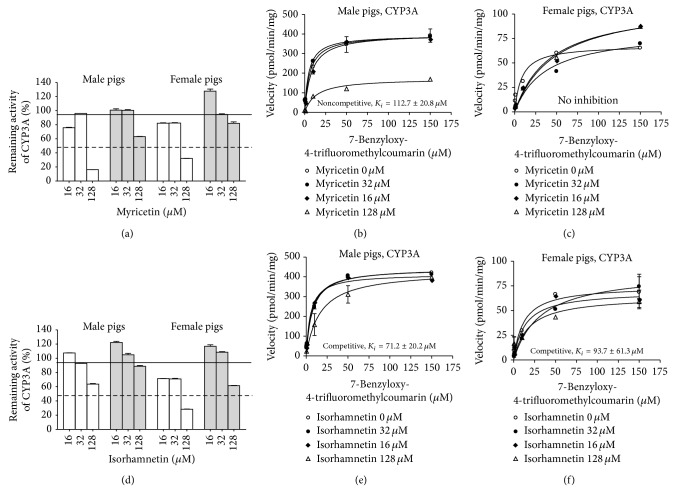
*In vitro* inhibition of CYP3A by myricetin and isorhamnetin in hepatic microsomes from male and female pigs. Data are presented as the mean percentage of remaining activity and standard error of the enzyme activity for three pools with microsomes from 2 male pigs in each pool and two pools with microsomes from 2 female pigs in each pool. (a) Effect of myricetin with (grey bars) and without (white bars) 5 min preincubation. ((b) and (c)) Saturation curve for 7-benzyloxy-4-trifluoromethylcoumarin O-dealkylation in hepatic microsomes from male (b) or female (c) pigs in the presence of myricetin. (d) Effect of isorhamnetin with (grey bars) and without (white bars) 5 min preincubation. ((e) and (f)) Saturation curve for 7-benzyloxy-4-trifluoromethylcoumarin O-dealkylation in hepatic microsomes from male (e) or female (f) pigs in the presence of isorhamnetin.

Of the tested compounds, the presence of myricetin and isorhamnetin decreased the CYP3A activity (Figure 1(c)). This inhibition was not altered by a preincubation step indicating reversible inhibition (Figures 3(a) and 3(d)). Kinetic analysis showed that myricetin inhibited CYP3A activity in a noncompetitive manner in male but not in female pigs (Figures 3(b) and 3(c)). Isorhamnetin acted as a competitive inhibitor of CYP3A activity in both male and female microsomes (Figures 3(e) and 3(f)).

**Figure 4:**
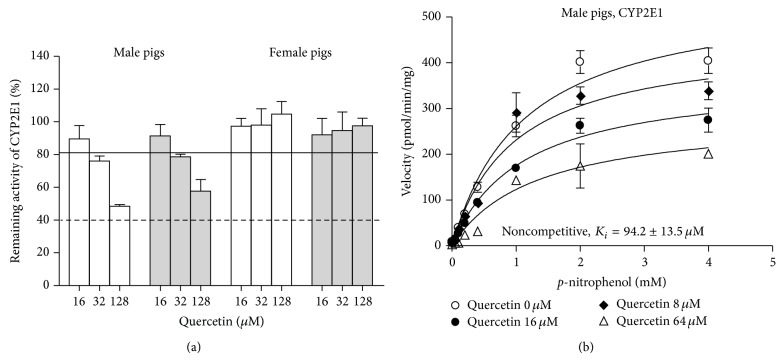
*In vitro* inhibition of CYP2E1 by quercetin in hepatic microsomes from male and female pigs. Data are presented as the mean percentage of remaining activity and standard error of the enzyme activity for three pools with microsomes from 2 male pigs in each pool and two pools with microsomes from 2 female pigs in each pool. (a) Effect of quercetin with (grey bars) and without (white bars) 15 min preincubation. (b) Saturation curve for* p*-nitrophenol hydroxylation (CYP2E1) in hepatic microsomes from male pigs in the presence of quercetin.

Among the tested compounds, quercetin inhibited CYP2E1 activity (remaining activity varied from 65.1 to 66.9%). This inhibition was observed only in the microsomes from male pigs (Figure 1(d)) and was not affected by preincubation step indicating reversible inhibition (Figure 4(a)). Analysis of the inhibition mode indicated noncompetitive inhibition of CYP2E1 activity (Figure 4(b)).

## 4. Discussion

In the present study, we investigated the interaction between several flavonoids commonly found in plants and the activity of the major drug-metabolizing CYP450 isoforms.

An important finding of this study is the gender-related differences in CYP450 response to flavonoids. Previously, we showed that CYP2E1 activity was inhibited by testicular steroids in male but not female pigs [4, 16]. Here, we demonstrated a similar effect of the flavonol quercetin, which inhibited CYP2E1-dependent activity only in microsomes from male pigs. Although CYP1A activity was inhibited by quercetin in both male and female pigs, the mode of inhibition differed, being competitive in male and mechanism-based in female pigs. Our results strongly suggest that regulation of CYP450 activities differs between genders within the same species. The significance of these findings is that gender-related differences should be taken into account when using porcine CYP450 as a model for studying food-drug interactions in humans. It should be noted that our study used the microsomes from 6 male and 4 female pigs, which might be considered as a limitation. Nevertheless, as all pigs serve as their own controls, we believe that our results provide important information on gender-related differences in CYP450 response to flavonoids.

Flavonoids have been found to modulate activities of at least four CYP450 isoforms of importance for xenobiotic metabolism: CYP1A1, 1A2, 1B1, and 3A4 [17]. The magnitude and mode of modification (induction or inhibition) were dependent on the specific flavonoid and its concentration. In rat hepatic microsomes, high concentrations of green tea flavonoids inhibited CYP1A (EROD) activity, although, at lower concentrations, they activated the same enzyme [18]. Data on CYP3A4 regulation by flavonoids is also contradicting. Quercetin induced CYP3A4 mRNA in primary cultures of human hepatocytes [19] and inhibited CYP3A4 activity in human liver microsomes [20] and human CYP3A4 expressed in Baculovirus-insect cell [21]. It was suggested that quercetin can act as both inhibitor and inducer of CYP3A4. An increased CYP3A4 activity in the presence of flavonoids has also been observed by Hosea et al. [22] and Atkins et al. [23]. This complex relationship between dietary flavonoids and CYP450 activity might cause unpredictable consequences with respect to Phase I metabolism of drugs and so forth.

Regulation of CYP450 activity by flavonoids can be the outcome of several events. They can act as agonists or antagonists of the receptors regulating CYP450 expression [2] or they can directly interact with the CYP450 enzymes as modulators of the kinetic parameters. Moreover, they can be substrates for the specific CYP450 enzymes changing probe substrate metabolism. In the present study, we showed that specific flavonoids can directly interact with the metabolic activity of several porcine CYP450 isoforms. In most cases, the effects were most pronounced for myricetin, quercetin, and isorhamnetin, compared to the other tested compounds. We have earlier found that inclusion of a preincubation step affects the inhibiting activity of steroids on CYP2E1 and CYP1A activity [4, 10]. Similar results were obtained in the present study. The inhibition of CYP3A by quercetin in female pigs increased when the preincubation step was included, suggesting irreversible or mechanism-based inhibition. This increase in the degree of inhibition might be due to formation of new hydroxymetabolites during preincubation step which are more potent inhibitors than the parent compound. Previous studies suggested that flavonoids possessing hydroxyl groups inhibit CYP3A activity, whereas those lacking hydroxyl groups can stimulate the enzyme activity [6, 20, 24]. Our results are in accordance with these observations. Myricetin, which has an extra hydroxyl group in the 5′-position compared to quercetin, had a strong inhibitory effect on CYP3A activity, whereas quercetin did not affect CYP3A. There is also a difference between the quercetin and its glucoside rutin in the effect on CYP1A and CYP2E1 activity; this may be explained by the glucosidation to rutinose via the 3-hydroxyl group, which inactivates the inhibitory capacity of quercetin. However, this needs to be further investigated. Moreover, quercetin has been determined to inhibit the metabolism of aryl hydrocarbons and to stimulate the activity of human CYP1A2 [25]. Thus, flavonoids can either inhibit or activate human CYP450s depending upon their structures, concentrations, and experimental conditions, and this occurs in a gender-dependent manner.

## 5. Conclusion

The data reported in this study for the first time suggest that the* in vitro* effects of specific flavonoids on porcine CYP450 activity are gender-dependent. The flavonoids myricetin, isorhamnetin, and quercetin were the most potent inhibitors of the major CYP450 isoforms. It is suggested that the degree of inhibition was dependent on flavonoid structure, flavonoid concentration, and the gender of pig. The phenolic acids (gallic, caffeic, and* p*-coumaric) and the flavonoid rutin did not alter activity of measured CYP450 isoforms. Although* in vitro* effects do not always predict* in vivo* situations, the results provide further insights into the mechanism of interactions of herbal components with drugs. Further studies are necessary to assess the significance of gender-related differences in flavonoid interactions with CYP450.
